# Mesenchymal stromal cells in the development and therapy of bronchopulmonary dysplasia

**DOI:** 10.1186/s40348-016-0046-6

**Published:** 2016-05-03

**Authors:** Marius A. Möbius, Mario Rüdiger

**Affiliations:** Department of Neonatology and Pediatric Critical Care Medicine, Medical Faculty and University Hospital Carl Gustav Carus, Technische Universität Dresden, Fetscherstrasse 74, Dresden, 01307 Germany; DFG Research Center and Cluster of Excellence for Regenerative Therapies (CRTD), Technische Universität Dresden, Fetscherstrasse 105, Dresden, 01307 Germany; Sinclair Centre for Regenerative Medicine, Sprott Centre for Stem Cell Research, Ottawa Hospital Research Institute, University of Ottawa, 501 Smyth Road, Ottawa, ON K1H 8L6 Canada

**Keywords:** Bronchopulmonary dysplasia, Lung injury, Stem cells, Mesenchymal stromal cells, Cell therapy, Newborn, Prematurity

## Abstract

Bronchopulmonary dysplasia (BPD), the chronic lung disease of prematurity, remains a major healthcare burden. Despite great progresses in perinatal medicine over the past decades, no cure for BPD has been found. The complex pathophysiology of the disease further hampers the development of effective treatment strategies, but recent insights into the biology of mesenchymal stem (MSCs) and progenitor cells in lung development and disease have ignited the hope of preventing or even treating BPD. The promising results of pre-clinical studies have lead to the first early phase clinical trials. However, these treatments are experimental and much more needs to be learned about the mechanism of action and manufacturing of MSCs. In this mini review, we briefly summarize the role of resident and exogenous MSCs in the development and treatment of BPD.

## Introduction

Premature birth and its associated pathologies remain one of the major causes of morbidity and mortality in children. Bronchopulmonary dysplasia (BPD) is the most common complication of birth before the 28th week of gestation and morphologically characterized by an arrest of alveolar growth along with a simplified pulmonary vasculature [1–3]. The development of the disease is complex and includes pre- and postnatal factors like preeclampsia, inflammation, malnutrition, and the exposure of the immature lung to relative hyperoxia and mechanical stress ex utero [1, 2].

The arrest of postnatal distal lung development is the hallmark of the so-called new BPD [2]. This suggests a role of stem or progenitor cells, the building blocks of growth, development, and regeneration, in the pathogenesis of BPD. Besides endothelial and certain epithelial stem cells [4], precursor cells from the mesenchymal lineage—mesenchymal stem (MSCs)—are thought to be involved in this process [5].

MSCs are fibroblast-like shaped cells that reside in virtually all mesodermal tissues of the fetal and adult body—including the bone marrow, adipose tissue, and lung—as well as in the placenta and the umbilical cord stroma [5, 6]. Essential features of MSC are

- Their ability to adhere to plain, uncoated plastic surfaces used in cell culture procedures
- The expression of CD73, CD90, and CD105 on the cell’s surface
- Absence of CD34, CD45, CD14/CD11b, CD19/ CD79 *α*, and HLA-DR
- The potency to differentiate along adipogenic, osteogenic, and chondrogenic lineages in vitro

Due to very low levels of expressed HLA class I and II molecules, MSCs are immune-privileged and do not trigger an immune response once administered to animals or humans in an allogeneic setting [7, 8]. This led to a large amount of clinical studies successfully utilizing the pro-angiogenic, anti-inflammatory, or tissue-regenerative potential of MSCs in adults [9].

Moreover, MSCs have been shown to effectively ameliorate experimental BPD when administered in a preventive or therapeutic approach [10]. These observations resulted in piloting clinical trials, where MSCs from the umbilical cord blood were administered to infants at risk for BPD [11]. Larger studies evaluating the clinical feasibility of this treatment are underway.

Nevertheless, the actions of the endogenous and therapeutically active exogenous MSCs are not well understood. In here, we briefly review the role of MSCs in the pathogenesis and treatment of BPD.

## MSCs orchestrate normal lung development

The lung is a complex organ deriving from the endodermal (→ epithelium) and mesodermal (→ mesenchyme, endothelium) germ line. It harbors several stem or progenitor cell types from these lineages, each of them with specific characteristics and (proposed) roles in lung development and regeneration [4].

Early normal lung development depends on the airway branching with subsequent formation of epithelial buds (pseudoglandular stage), their narrowing (canalicular stage) and septation into sacs and the alveolar precursors (saccular stage, *primary septa*). Subsequent remodeling and thinning of the mesenchymal compartment results in a convergence of capillaries and lung epithelium and triggers septation of the sacs into mature alveoli (alveolar stage, *secondary septa*). This dramatically increases the alveolar surface of the lung and ensures easy gas exchange between blood and air. In humans, alveolarization starts at approx. 32 weeks of gestation and continues until the fifth year of life [3, 12]. This process is disturbed in BPD, leading to the increased oxygen demand defining BPD [2].

The lung’s mesenchyme—consisting of MSCs, fibroblasts, and their secreted extracellular matrix (ECM)—crucially drives and modulates lung development [2, 3, 12, 13]. Especially alveolar myofibroblasts, along with the mesenchymal products elastin, fibroblast growth factors 9 and 10 (Fgf9, Fgf10), Wnt, and platelet-derived growth factor alpha (Pdgf *α*) are involved in normal lung development by enabling both early epithelial budding and subsequent alveologenesis by formation of secondary septa [12, 13].

Lung-MSCs (L-MSCs) represent a diverse population of organ-resident mesenchymal progenitors [5, 14]. They have been shown to exhibit a highly lung-specific gene expression pattern and secrete extensive amounts of proteins contributing to lung development, including the abovementioned ones. Moreover, L-MSCs are thought to give rise to several other lung mesenchymal cells like myofibroblasts, lipofibroblasts, and normal lung fibroblasts during early development and organ maturation until adulthood [5].

Functional experiments substantiated the role of L-MSCs in normal lung development. In contrast to stem cell antigen-1 (Sca-1)-positive/CD90-negative lung (myo-)fibroblasts, Sca-1 ^pos.^/CD90 ^pos.^ L-MSCs support the growth of epithelial progenitors like alveolar epithelial type II cells (AEC-II) and Clara cells in vitro [15]. Lipofibroblasts, proposed descendants of L-MSCs, have been shown to be involved in early surfactant production by transferring triglycerides (TG) to AEC-II. The epithelial cell triggers this reaction by secretion of prostaglandin E2 and parathyroid hormone-related protein (Pthr-p). AEC-II further metabolize the received TG—additionally stimulated by lipofibroblast-derived leptin—to surfactant (reviewed by Collins/Thébaud [5] and El Agha/Bellusci [15]). Moreover, co-culture of AEC-II and adult lipofibroblasts leads to the formation of small alveolar-like structures (alveolospheres) in vitro, indicating the vital role of mesenchymal-epithelial crosstalk in postnatal lung development and regeneration [16]. Lipofibroblasts further store and secrete retinolic acids, important mediators of alveolar septation, and lung development [15].

Striking evidence for the crucial role of lung mesenchymal progenitors in normal lung development was recently provided by Dr. Krasnow’s group. Utilizing a sophisticated single-cell labeling in the rodent lung, Kumar et al. described a diversity of stem/progenitor cell populations within the mesenchyme. Each MSC population exerted distinct lineage and migration boundaries and contributed to the formation of stalk mesenchyme, mesothelium, and smooth muscle surrounding the developing airways or pulmonary vasculature. Moreover, the group demonstrated that labeled progenitors of airway smooth muscle cells orchestrated lung morphogenesis by inducing differentiation of stalk mesenchymal cells into airway smooth muscle cells during epithelial budding and branching morphogenesis. Epithelial signaling alone was unable to induce this essential process [14]. These results suggest that L-MSCs do not only act as progenitors of mature mesenchymal cells but also as “control center” of lung development by coordinating the fate of other cell types.

Nevertheless, the MSCs’ hierarchy and function within the mesenchymal compartment of the lung seems to be very complex. Further research will focus on elucidating the pathways of L-MSC differentiation and mechanisms of interaction with epithelial and endothelial lung cells.

## Disrupted mesenchymal development and impaired lung maturation

As mentioned previously, normal lung development depends on the well-orchestrated interaction between mesenchymal, epithelial, and endothelial cells. In BPD, this process is disrupted, leading to the characteristic arrest of alveolar and capillary development [2, 3].

The observation that the presence of lung-specific MSCs in the tracheal aspirate of premature newborns predicts the subsequent development of BPD [17] focused research on the role of these cells in the pathogenesis of the disease. The MSCs isolated from infants who later developed BPD demonstrated substantial alterations in the pathways controlling the differentiation towards myofibroblasts—platelet-derived growth factor (PDGF)-receptor- *α*, *β*-catenin, and TGF *β*1—in vitro [5, 18]. Disruptions in these pathways have been described to lead to interstitial fibrosis and reduced formation of secondary septa, consistent with the histopathological presentation of BPD [18]. However, the relationship between the MSCs present in the tracheal aspirate and the tissue-resident L-MSC is not clear and subject of further research.

As the lung matures in a hypoxic environment in utero, postnatal (relative) hyperoxia represents a potent stressors to the immature lung and leading cause of BPD [2]. Hyperoxia triggers inflammatory processes in the lung which results in aberrant mesenchymal signaling, especially along the Fgf-10 pathway [13]. Moreover, disrupted mesenchymal PDGF *α*/PDGFR- *α* signaling was observed in neonatal rodent lungs subjected to 75 % O2 [18]. Both processes led to disrupted alveolar development.

L-MSCs isolated from human fetal lungs demonstrate profound alterations in their phenotype and secretory behavior once cultured in normoxic (21 % O2) and hyperoxic (60 % O2) atmospheres, including rapid differentiation into pro-fibrotic myofibroblasts and reduced production of elastin and certain other ECM components [5]. Elastin is a crucial component of the lung-ECM, which acts as structural protein and driver of—especially alveolar—lung development. It is known that quantitative impairment of lung elastin, i.e., in *Eln*^−/−^ mice leads to arrested terminal airway formation. Moreover, structural abnormalities and disrupted deposition patterns of ECM components have been described in BPD [2, 13].

**Fig. 1:**
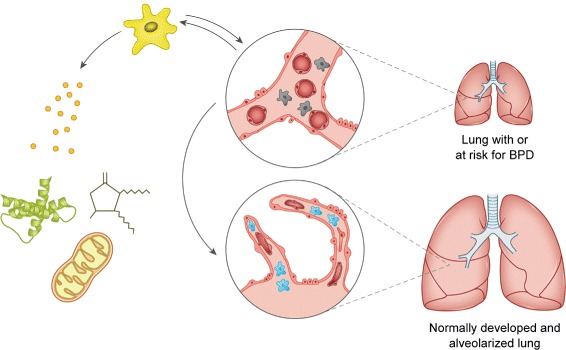
Hypothesis of the MSCs action in BPD. Exogenous MSCs (*yellow*) interact with the immature lung tissue (*upper lung schema* diseased endogenous lung MSCs gray, thick mesenchymal layer forming the primary septa, containing two layers of capillaries (*red*) distant from the epithelium) and secrete exosomes and cytokines and transfer mitochondria (left handed site). This protects and rescues lung development, driving the maturation of the mesenchyme with healthy endogenous MSCs (*blue*), formation of secondary septa and thinning of the blood-air barrier (*lower lung schema*)

In summary, it can be hypothesized that oxygen—along with other deleterious factors damaging the immature lung—impairs L-MSCs. These cells may not be able to differentiate properly, giving raise to aberrant cell types like stress-induced myofibroblasts [5]. Further differentiation and loss of the MSCs’ function to coordinate lung growth, along with disrupted ECM and cytokine production then leads to the failure of the lung to mature and develop postnatally. This hypothesis (Fig. 1) is difficult to prove, given the diversity of L-MSC populations and the resulting complicated methods to label and track them [14]. But the observation that exogenous MSCs prevent and rescue BPD strongly supports this concept.

## Exogenous MSCs prevent and rescue disrupted lung development

MSCs have been successfully investigated in a vast number of preclinical and clinical studies; so far, over 1000 patients have safely been treated with these cells for cardiovascular, neurological, hemato-oncological, or autoinflammatory disorders [9]. Different concepts have been developed to utilize the beneficial effects of MSCs: (I) the application of whole cells, (II) the administration of cell-free concentrated tissue culture supernatants (“conditioned medium” or CdM) containing the MSCs’ secreted factors, or (III) the usage of purified, defined components of these supernatants, i.e., exosomes, small vesicles containing protein, and microRNA.

The regenerative, anti-inflammatory, and pro-angiogenic potential of MSCs [6] derived from the bone marrow (BM-MSCs), umbilical cord blood (UCB-MSCs), or the Wharton’s jelly (UC-MSCs) predestinate them for a therapeutic use in experimental BPD. Most of these studies have been performed in a model of rodent hyperoxia-induced lung injury, which mimics most of the morphological and functional features of the “new” BPD. This includes alveolar and capillary simplification, lung fibrosis with disordered ECM deposition, pulmonary hypertension with vascular remodeling, and an influx of inflammatory cells [19]. Using this model, the group led by Dr. Thébaud demonstrated that intratracheal administration (i.t.) of BM-MSCs prevents the offset of all these pathophysiological changes [20]. Simultaneously, Aslam et al. described comparable effects in mice treated intravenously with either BM-MSCs or their CdM in a preventive approach [21]. MSCs are also capable of (partially) rescuing experimental BPD, which was demonstrated utilizing intratracheal administration of UCB-MSCs. Using these cells in a preventive approach, improvements in lung architecture, pulmonary hypertension, and exercise capacity persisted until adulthood (6 months) of the rats. [22]. Engraftment of MSCs was surprisingly low in all of these studies, indicating a mainly paracrine action rather than an integration and differentiation into lung cells. These promising reports led to several studies investigating the effects of MSCs or their CdM from various sources in experimental BPD. All of them found comparable and consistent beneficial effects on the lung architecture and function [10, 23].

The paracrine effects of MSCs are predominant in BPD, as indicated by the effective usage of cell-free CdM [23]. Blunting of inflammation—a crucial component of BPD pathogenesis—seems to account for the immediate beneficial effects. Mainly involved cytokines are interleukin-10 and TNF-stimulated gene/protein 6, which both act strongly anti-inflammatory [8]. Moreover, MSC-derived proteins like stanniocalcin-1 seem to prevent damage to the endogenous (progenitor) cells by increasing their resistance to oxidative stress [24, 25]. Secreted growth factors like hepatocyte growth factor (HGF) and vascular endothelial growth factor (VEGF) then stimulate lung development. MSCs also produce exosomes, small membrane-coated vesicles containing lipids, proteins, and microRNA. Especially, the microRNA can interfere with the transcription of certain genes, thereby causing the up- or downregulation of growth factors in lung cells [10].

However, some authors described that treatment with CdM fails to cause long-term effects comparable to whole cell therapy [26], whereas short-term effects of CdM are equal or even superior [21] to an administration of whole cells (reviewed by Fung et al. [23]). A direct interaction of the exogenous MSC with the damaged lung seems to potentiate their therapeutic efficacy in some cases. Several hypotheses exist to explain this phenomenon: It is known that—besides paracrine effects—MSCs are capable of modulating inflammation by direct cell-cell contact with leukocytes [10], which may result in a higher anti-inflammatory potency of whole cell therapy. Secondly, MSCs are thought to act as part drug and part device that senses, integrates, and responds to its surrounding with exactly the quantity and quality of therapeutically active substances needed—an eventually “intelligent and personalized” therapeutic approach [27]. Moreover, reconstitution of impaired ATP metabolism by mitochondrial transfer from MSCs to lung cells via specific channels has been described in acute lung injury [28]. This could occur as well in the treatment of BPD, causing a persistent therapeutic effect by permanently transplanted mitochondria.

## MSCs in infants—are we there yet?

The promising preclinical results led to a piloting clinical study utilizing UCB-MSCs in infants. Chang and colleagues used 1 × 10^7^ or 2 × 10^7^ MSCs per kg in nine premature infants born between 24 ^+0^ and 26 ^+4^ weeks of gestation. The cells were administered i.t. between the 5th and 14th day of life without signs of dose-limiting toxicity or severe adverse effects. As compared to an age-matched control group, a trend towards a lower BPD severity was noted [11]. Several phase 1 and phase 2 trials are underway (NCT02443961, NCT02381366, NCT01828957).

However, using MSCs in our most vulnerable patient population might be—despite of the euphoria around their healing capacities and the great-looking preclinical reports—a bit ahead of the science. Besides smaller issues which can be targeted in clinical trials (patient selection, administration route, dosing, timing of administration), one major obstacle hampers the initiation of large, controlled trials using MSCs in infants and other patients: No standardized, high-quality cell product is available to test in clinical studies. Many laboratories and companies developed (and patented) methods to isolate and expand MSCs from various sources [6, 29]. But no quality control, method of standardization, or method to determine the therapeutic activity of an MSC-based cell product exists. This is of particular interest as all production procedures (isolation, expansion, passaging, harvesting) influence the MSC. This causes differentiation and other cellular impediments affecting the activity of the final product, regardless if whole cell, CdM, or purified exosome preparations are considered [29]. Without quality control prior to administration, results from studies—even when conducted with the same cell type—are not comparable. Large trials are difficult to initiate and their results difficult to interpret and compare. Intensive translational research is required to overcome this issue, and afterwards, clinical studies will determine not only which therapeutic approach is the most safe, effective, and efficacious but also which target population of infants benefits most from MSCs.

## Conclusions

L-MSCs orchestrate lung cell growth and therefore play an important role in normal development. Damage to endogenous MSCs may occur in BPD and contribute to its pathogenesis. Exogenous MSCs harbor the fascinating potential to specifically target several mechanisms involved in BPD development. Their potential to protect and regenerate lung cells does not only prevent but also rescues lung injury at least in animal experiments. Promising preclinical data warrants piloting clinical studies, but current cell products are imperfect and hamper the initiation of large, pivotal trials. Every novel technology is flawed at the beginning, and trials in patients seem to answer many questions that cannot be addressed in the laboratory. But it is imperative to due diligence and treat our most vulnerable patient population with the best possible cell product in order to obtain the best possible rather than the fastest results. Further basic and translational research, especially concerning the better characterization and quality control of cellular therapeutics is required before carefully designed clinical trials can be initiated. With this, cellular therapeutics like MSCs can become the next game changers in neonatal pulmonary care.
